# Real-time and stepwise deoxidization processes to tune the photoluminescence properties of graphene oxide using EC-SPR spectroscopy

**DOI:** 10.1039/c7ra13594g

**Published:** 2018-03-23

**Authors:** Nan-Fu Chiu, Cheng-Du Yang

**Affiliations:** Laboratory of Nano-photonics and Biosensors, Institute of Electro-Optical Science and Technology, National Taiwan Normal University Taipei 11677 Taiwan nfchiu@ntnu.edu.tw

## Abstract

The development of a stepwise deoxidized process and real-time monitoring of the large-scale mass production of electrochemically reduced graphene oxide (ErGO) sheets are important issues. In this study, we have shown that graphene oxide (GO) sheets can be quantitatively monitored in real-time and controlled in a stepwise manner using electrochemical-surface plasmon resonance (EC-SPR), due to the fact that the oxygen functional groups can be tuned through a deoxidization procedure. The SPR signal can then be detected quantitatively in real-time by changes in the dielectric constant of the GO film during the EC stepwise removal of oxygen functional groups. This is because the refractive index of the GO sheets is affected by the oxygen-containing groups, so that monitoring the SPR angle shift provides a real-time measure of changes in the concentration of the residual oxygen functional groups of the GO sheets. In this study, we demonstrated GO and 100 CV cycles of ErGO at X-ray photoelectron spectroscopy carbon-to-oxygen ratios of 4.1 and 31.57 respectively, and Raman spectra of the D/G intensity ratio of 0.85 and 1.89, respectively. The 100 CV cycles of ErGO at SPR angle shifts were −227.13 mdeg for GO at a concentration of 0.275 mg ml^−1^, and −263.47 mdeg for GO at a concentration of 1 mg ml^−1^. The photoluminescence emission bands of the GO and the CV 100 cycles of ErGO were 615 to 470 nm. These results may be beneficial for future studies on GO fluorescence characteristics in the field of optoelectronic and biosensor applications.

## Abstract 2

The development of a stepwise deoxidized process and real-time monitoring of the large-scale mass production of electrochemically reduced graphene oxide (ErGO) sheets are important issues.

## Graphical abstract

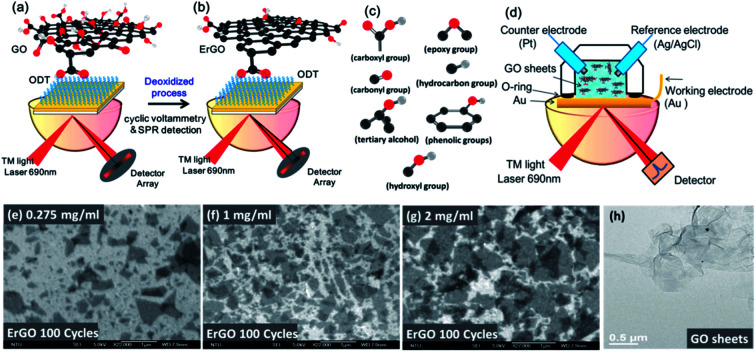

## 1. Introduction

Graphene oxide (GO) has abundant oxygen-containing functional groups, a natural fluorescence band, and many beneficial properties including low cost, nontoxicity, biocompatibility, and being environmentally friendly. The oxygen-containing functional groups include epoxy, hydroxyl, carbonyl, and ether groups on the graphene surface, and they can modulate the photoluminescence (PL) emission spectra^1–3^ through the recombination of electron holes between the conduction band and the valence band, which provides an opportunity to tune the conductivity^4,5^ and stress properties.^6^ Extensive research has been conducted to investigate this issue because the oxygen functional groups can be readily processed. Chemical modification,^1,7,8^ laser,^9^ photothermal,^10,11^ thermal exfoliation,^7,12^ ozone,^13^ and electrochemical^1,14^ methods have been widely used to reduce GO sheets and to modulate the GO fluorescence spectrum. GO sheets have also demonstrated absorbance in the ultraviolet (UV) region from 200 to 300 nm, which is related to π–π* and π–n* electron transitions.^15^ GO sheets have a PL emission in the visible (vis) broad band range from 400 to 800 nm. The changes in luminescence in wavelength and intensity of the excited GO sheets may be related to σ*–n, π*–π, and π*–n electron transition bonds (belonging to electron transitions between antibonding and bonding molecular orbitals) between the oxygen functional group and the carbon structural material.^10,15,16^ This luminescence has been reported to be related to three oxidative functional groups C–O, C=O and O=C–OH, which have a close relationship and are involved in the reaction,^10,15^ leading to various applications including biosensors,^17–20^ and fluorescence spectra.^21^ In addition, GO sheets can be used as effective fluorophores^10,14,22,23^ and in quenching^24,25^ to enhance the efficiency of the excitation energy transfer (Förster) process, and this has been widely used in advancing biosensors for cell imaging applications. Moreover, the GO surface has residual oxygen-containing functional groups, which may lead to poor crystallinity with no uniformity.^26,27^ Other applications of reducing GO to reduced graphene oxide (rGO) film-based carbon materials include increasing the stability of electrodes,^28^ improving charge carrier mobility,^7,29^ tuning its dielectric and optical band-gap properties,^1,10,14^ enhancing binding interactions and improving biosensor sensitivity,^30,31^ modulating surface plasmon resonance (SPR) energy,^32,33^ increasing the efficiency of gas molecule adsorption^34,35^ and drug delivery loading,^36^ all of which can contribute to advances in science and technology. We previously demonstrated the first electrochemical-surface plasmon resonance (EC-SPR) immunosensor incorporating an electrochemically reduced graphene oxide (ErGO) film, and showed the potential of the specific affinity properties of ErGO in electrochemical-enhanced biosensing.^37^

In this study, we propose an alternative method to chemical and thermal reduction by simultaneously using SPR real-time monitoring of changes in the GO deoxygenation process and electrochemical (EC) stepwise reduction of GO to ErGO. This EC-SPR technique can monitor and control the oxygen-containing functional group in a stepwise manner on the GO surface in real time, and can improve GO surface crystallinity defects, as GO surface crystallinity defects are closely related to the preparation methods and reduction technologies depending on the size of the GO sheet layers. These phenomena will directly affect the characteristics of the fluorescence emission. There are currently no relevant studies on the use of the EC-SPR technique for the real-time monitoring and stepwise reduction of GO fluorescence emission, and no discussion of the related issues. Our results show that EC-SPR could be successfully applied to the development of GO for the simultaneous detection of residual oxygen functionality in ErGO leading to changes in the refractive index (carbon-to-oxygen (C/O) ratio and band-gap) resulting in angle shifts. Using real-time monitoring and the stepwise reduction of GO are conducive to the future development of luminescent semiconductor GO materials and the future of sensing materials. More importantly, the emission spectra of GO sheets can effectively be tuned, and therefore have the potential to advance the field of fluorescence in various applications, as well as biosensing technology. Of these applications, assays for naked-eye biosensors are the most common due to their simplicity, rapid screening ability, semi-quantitative analysis, and low cost.

## 2. Experimental section

### 2.1 Materials

Graphite was purchased from Graphene Supermarket (Graphene Laboratories Inc., Reading, MA, USA). GO sheets were synthesized by using a modified Hummers' method^38^ followed by ultrasonic shattering for 5 hours to obtain a flake size of 0.1–1 μm, thickness of 1.1 nm. 1-Octadecanethiol (ODT, C_18_H_37_SH, 96%), sodium chloride (NaCl) and potassium chloride (KCl) were purchased from Sigma-Aldrich and used as received.

### 2.2 Preparation of ErGO chips and ErGO solutions

**Fig. 1:**
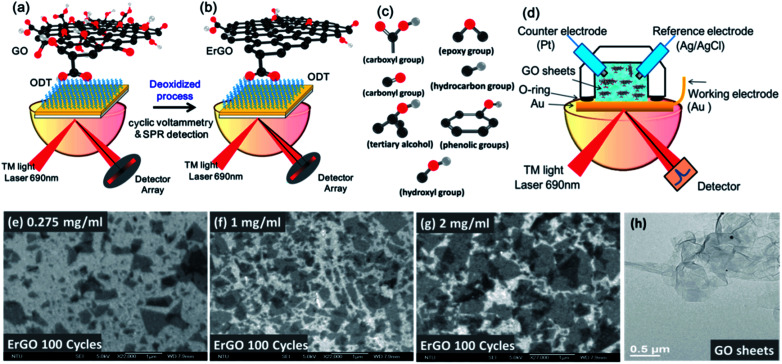
Schematic diagram of the EC-SPR setup for the *in situ* conversion of GO to ErGO films. (a) GO sheets were immobilized on a gold surface. (b) The deoxidization process of ErGO. (c) The oxygen functional group of GO. (d) The suspension of GO sheets in aqueous solutions in the EC-SPR real-time electrochemical reduction system. The SEM images at different concentration conditions under the deoxidized process of the ErGO film for (e) 0.275, (f) 1, and (g) 2 mg ml^−1^. (h) TEM image of GO sheets.

ErGO can be reduced in two different ways: reduction of GO chips and direct reduction of GO solution using an electrolyte. The first method uses GO sheets immobilized on the Au surface of electrodes using a self-assembled monolayer (SAM) technique.^17–20,37^ GO sheets were prepared by oxidation using Hummers' method to accomplish highly hydrophilicity and good dispersibility in a suspension of GO. We used the modification of SAMs of the ODT linker on a gold (Au) surface for 24 h. The GO sheets were then diluted to concentrations of 0.275 and 1 mg ml^−1^ in aqueous suspension, and immersed on an Au chip to immobilize the GO sheets for 5 h as shown in Fig. 1a. The EC-SPR signals were recorded during real-time deoxidization of the GO films on the Au electrode at a CV cycle scan rate of 50 mv s^−1^ in a 0.5 M NaCl solution, with a potential ranging from −1.1 to 0.7 V.

ErGO chips were obtained from GO chips electrochemically with different reduction conditions as shown in Fig. 1b. Adsorption of functional oxygen in the form of epoxy, hydroxyl, hydrocarbon, carbonyl, carboxyl and ether groups created in the GO sheets is shown in Fig. 1c.

In the second method, the GO solution-based reduction of GO was performed in phosphate buffered saline (PBS), NaCl and directly reduced with the aforementioned electrochemical method. ErGO solutions were prepared from the GO aqueous suspensions at different concentrations (0.01, 0.275, 1, and 2 mg ml^−1^) in a volume of 1 cm^3^ of the ErGO solution. In the ErGO aqueous suspensions, there was no need to link the GO sheets for immobilization on the Au surfaces, as shown in Fig. 1d. The scanning electron microscope (SEM) images of different concentrations of GO films after ErGO are shown in Fig. 1e–g. The transmission electron microscope (TEM) image showed that the GO sheets exhibited a configuration of a few 2D layers with a typical wrinkled flake structure (Fig. 1h).

### 2.3 Characterization

SEM images were obtained using a JEOL JSM-6700F field emission-SEM (FE-SEM) system, and TEM images were obtained using a 300 kV field-emission gun TEM system (Tecnai G2 F30 S-Twin; Philips-FEI). The X-ray photoelectron spectroscopy (XPS) experiments were performed using 24A1 and 09A2 beamlines at the National Synchrotron Radiation Research Center (NSRRC), Hsinchu, Taiwan.^39^ Fourier-transform infrared spectrometer (FTIR) measurements were made using a Bruker Vertex 80v spectrometer in attenuated total reflection (ATR) mode at the Instrumentation Center at National Tsing Hua University, Taiwan. To demonstrate the spectra changes at various GO and ErGO concentrations, transmittance spectra were obtained using a UV-vis spectrophotometer (U-2900, Hitachi High-Technologies Corporation, Japan) with a wavelength from 200 to 1100 nm at room temperature. Raman measurements were performed using a microscopic Raman system (MRI, Protrustech Co., Ltd., Taiwan) with a Mount Qic Demountable Laser (532 nm) as the excitation source, with the laser power below 10 mW to avoid laser-induced heating. The MRI system provided very steep transitions from 90 cm^−1^. Raman measurements were performed in a back scattering configuration on a micro-Raman system equipped with an air-cooling spectrometer (AvaSpec-ULS2048L) with a grating of 1800 lines per mm and slit of 50 μm as the detector. The EC-SPR measurements were performed using a BI-3000G SPR Instrument (Biosensing Instrument Inc., USA), which enabled the real-time monitoring of the index of refraction at a resolution of <10–8 units, and angular modulation down to <10–5 degrees for a 690 nm wavelength light source.^40^ The electrochemical reduction of the GO films was performed in a 2 ml internal sample volume cell using a CHI-604D electrochemical analyzer work station (CH Instruments Inc., Austin, TX, USA) for the three-electrode system including the modified Au electrode as the working electrode, platinum (Pt) wire as the counter electrode, and Ag/AgCl (saturated KCl) as the reference electrode. PL measurements were performed using a 405 nm diode laser at 30 mW (Tayhwa Technology Co., Ltd., Taiwan) and a high resolution spectrometer (HR 2000+, Ocean Optics, Inc., USA) at a fixed incident angle for the normal (0°) angle and spectrometer at 45°.

## 3. Results and discussion

### 3.1 Analysis of EC with SPR properties of the GO and ErGO films

**Fig. 2:**
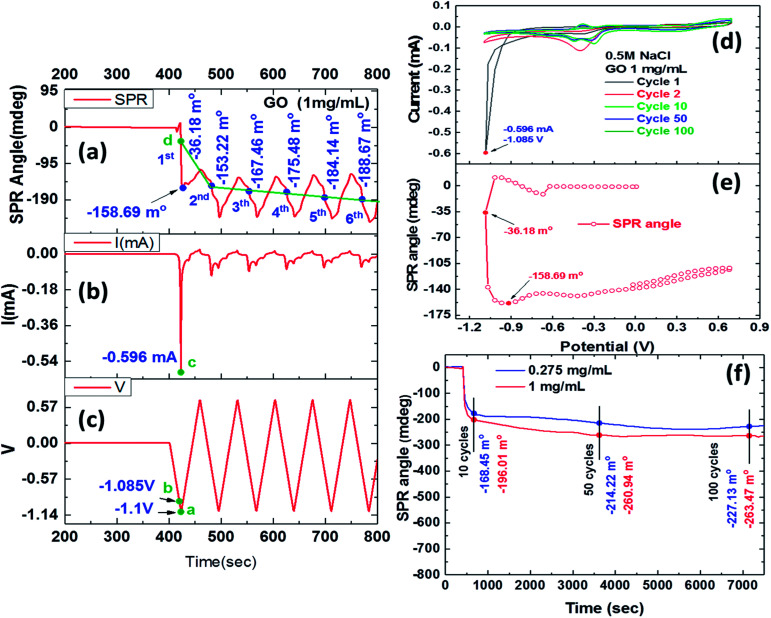
The real-time deoxidization of GO films in the EC-SPR study of current and SPR angle responses during cyclic voltammetry scans (six cycles) in 0.5 M NaCl solution, with the potential ranging from −1.1 to 0.7 V *versus* SCE and a scan rate of 50 mv s^−1^: (a) SPR angle (b) current; (c) potential. Cyclic voltammograms showing GO redox peaks at; (d) current *versus* redox potential and the (e) SPR response, verifying the GO film deoxidization process. (f) Relationship between real-time SPR angle shifts of CV cycles and redox reaction time in the deoxidization process of the GO films, yielding oxygen-containing functional group in ErGO after ten CV cycles, 50 CV cycles, and 100 CV cycles.

Fig. 2 shows the reduction process of 1 mg ml^−1^ GO films by the EC-SPR curve reaction characteristics. The SPR angle shift due to the reduction process showed an obvious shift during the first cycle and significant stepwise changes in the next six cycles as shown in Fig. 2a. GO appears to be reduced to ErGO through the deoxidized process after the first cycle with the electrochemical behaviour of the obtained ErGO and SPR angle shift of −36.18 mdeg. The SPR shift in this CV scan result showed that the second, third, fourth, fifth and sixth cycles were −153.22, −167.46, −175.48, −184.14 and −188.67 mdeg, respectively. In the conductive electrolyte solution, the instability of the real-time SPR angle curve may have been caused by an instantaneous double layer interface charge density, occurring almost instantaneously in response to a potential perturbation in the dielectric properties of the reduction of GO.^41–44^

Fig. 2b and c show the CV curve of the reaction current and proper procedure potential of a triangular wave, respectively. Fig. 2b shows a GO reduction peak of the current curve at −0.596 mA for point “c”, and Fig. 2c shows an electrochemical reduction voltage of GO of around −1.085 V for point “b” during the first CV cycle. The first irreversible oxidation reaction exhibited a pronounced peak, showing a maximum current at a potential of 1.085 V. Fig. 2c shows that point “a” had a CV scanning potential of −1.1 V, and Fig. 2a shows that point “d” had an SPR angle shift of −36.18 mdeg in the first CV cycle. The potential started at 0 V, however, the GO reduction process resulted in a gradual decrease in the SPR angle shift in each cycle. As the instability of the SPR angle shift represents the changes of instantaneous double layer interface charge density, electrochemical oxidation and reduction currents may cause such a shift in response to a potential perturbation in the dielectric properties of the reduction of GO.^45^

Fig. 2d shows that the reduction current began to drop significantly in the first voltammetric scanning cycle, and that ErGO showed a lower reduction potential of −1.085 V, yielding a current peak at −0.596 mA. This result indicated that the ErGO film that was formed at −1.085 V had a lower reduction peak than that obtained in the first cycle of the electrochemical reduction. In later cycles, the negative shifting of the applied reduction potentials shrank the reduction peak of the resulting ErGO films. A reduction current was observed in the ErGO film that was prepared using a reduction potential of −1.085 V, showing the efficient stepwise electrochemical reduction of the oxygen groups under this condition. The reduction current continued to fall until it disappeared, and the deoxidization processes exhibited irreversible properties. The plots showed the relationship between the current and the shift in the SPR angle, as shown in Fig. 2d–f. The stepwise cyclic voltammograms and real-time SPR curves were recorded in the first CV cycle of the electrochemical redox-reaction. Fig. 2e shows the SPR angle shift and complete, stepwise deoxidization of GO. The first CV at a potential of −1.085 V increased the reduction current to −0.417 mA and generated an SPR angle shift of −101.9 mdeg. The SPR angle could be observed in the sharp reduction in the current and potential-dependent change in the deoxygenation process, and the largest shift was produced at an SPR angle (*θ*_SPR_) of −201.0 mdeg. In a related report, the deoxy-reduction of GO to ErGO reduced the thickness from 1.2 nm to 0.8 nm and increased the refractive index from 2.24 to 3.5. Therefore, the influence of the shift in the SPR resonance angle in the stepwise deoxygenation process that changed the refractive index was far stronger than that in the deoxygenation reaction that reduced the thickness of the 0.275 mg ml^−1^ ErGO film.^46,47^

Fig. 2f shows the real-time SPR evaluation of the deoxidization process at various scan cycles at a scan rate of 50 mv s^−1^. During several stepwise CV cycles, the oxygen groups in the GO were progressively reduced, and as the number of CV cycles increased, the C/O ratio increased and the number of residual oxygen functional groups decreased. The SPR angle shift that was caused by the reduction process in the first cycle was obvious. Fig. 2e shows the plot of SPR response curves that were obtained by real-time monitoring of the residual oxygen-containing functionality of the ErGO film and changes in the refractive index which caused an angle shift. The results showed that long-term monitoring of SPR angular displacement was obviously affected by environmental temperatures, resulting in gradual changes in the drift angle. Table 1 shows the SPR angle shifts upon electrochemical reduction for 10 (720 s), 50 (3600 s) and 100 (7200 s) CV cycles of the deoxidization process were −168.45, −214.22 and −227.13 mdeg for a GO concentration of 0.275 mg ml^−1^, and −196.01, −260.94 and −263.47 mdeg for a GO concentration of 1 mg ml^−1^, respectively.

**Table 1:** Relative intensities of XPS spectral peaks in the GO and ErGO films

Films	XPS spectra (O_1s_)		XPS spectra (C_1s_)						SPR (*θ*, mdeg)	
	O=C (%)	O–C (%)	C–C, sp^2^ (%)	C–C, sp^3^ (%)	C–O (%)	C=O (%)	O–C=O (%)	C/O ratio	0.275 mg mL^−1^	1 mg mL^−1^
GO	85.6	14.4	76.59	3.79	17.47	1.38	0.78	4.1	0	0
ErGO (10 cycles)	86.9	13.1	78.59	12.5	7.38	0.95	0.58	10.22	−168.45	−196.01
ErGO (50 cycles)	94.5	5.5	79.31	15.11	4.2	0.85	0.53	16.92	−214.22	−260.94
ErGO (100 cycles)	95.8	4.2	80.88	16.05	2.32	0.54	0.21	31.57	−227.13	−263.47

We previously used the multilayer reflection model theory for Fresnel's law to calculate the GO and ErGO film at the SPR angle shift (*θ*_sp_) *versus* thickness (*d*) to verify the calculated relationship between the refractive index and thickness of GO and ERGO at the SPR angle shift.^37^ These results suggest that the effect of the electrochemical reduction GO can effectively remove interlamellar water layers, resulting in an increased refraction index of ErGO and reduced oxygen content of sheet layers, which then results in a significant shift of the SPR angle in a real-time response.

The combination of SPR and electrochemical stepwise deoxidization process enabled large changes in SPR angle shifts as a result of reducing the oxygen content in the conductivity of the GO film on a gold electrode surface. The electrochemical stepwise deoxidization process changed the oxygenated functional groups of the GO sheets and reduced the number of oxygen bonds. This then led to an increase in the refraction index of the ErGO sheets, and possibly also resulted in a reduction in their thickness. Partial removal of intercalated water and oxygen may also have affected the measured thicknesses, thereby resulting in a significant shift of the SPR angle in a real-time response.

### 3.2 Analysis of optical properties of the GO and ErGO films

**Fig. 3:**
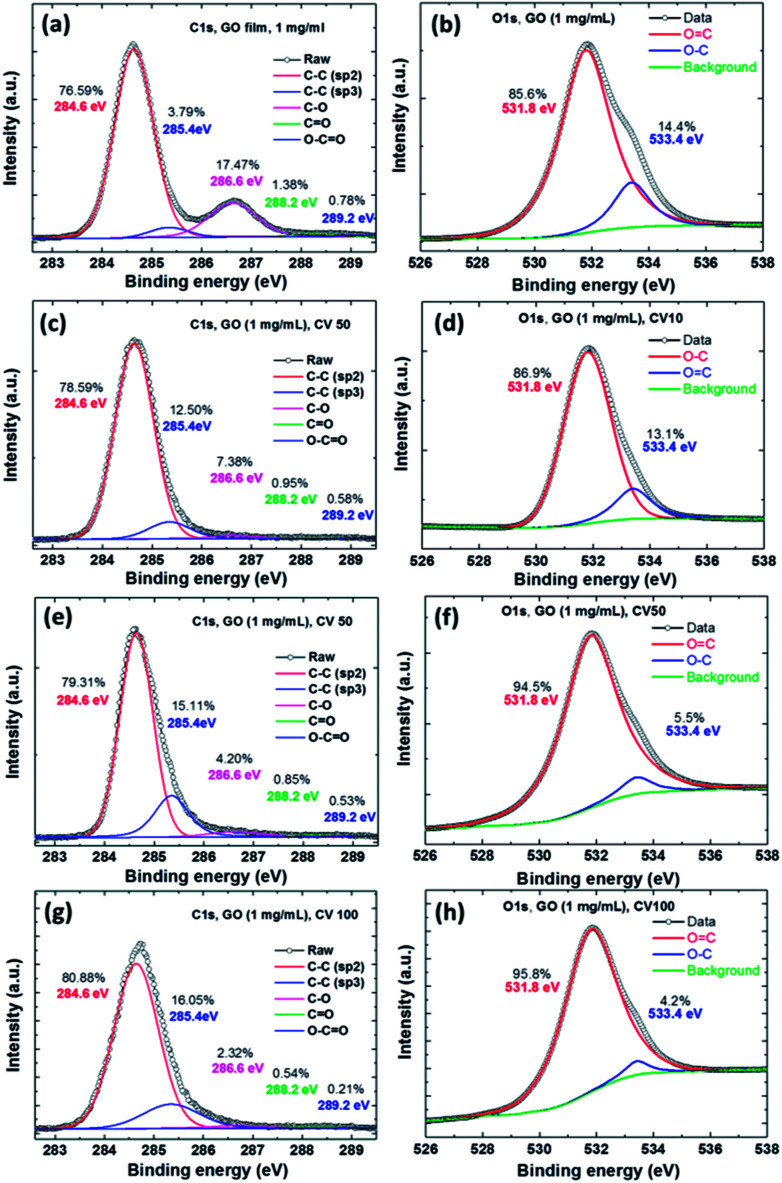
The C 1s and O 1s XPS spectra of the GO films at different electrochemical reduction cycles. (a and b) GO film, (c and d) ErGO film for 10 CV cycles, (e and f) ErGO film of 50 CV cycles, and (g and h) ErGO film of 100 CV cycles.

We used XPS to analyze differences in electrochemical reduction cycles of the GO films (Fig. 3). Fig. 3a shows the C 1s XPS spectra of a GO film, which clearly shows a considerable degree of oxidation with four components that corresponded to carbon atoms in different functional groups, including the non-oxygenated ring C–C for sp^2^ (284.6 eV, 76.59%), C–C for sp^3^ (285.4 eV, 3.79%), C–O bonds (286.6 eV, 17.47%), carbonyl C=O bonds (288.2 eV, 1.38%), and carboxylate carbon O=C–O bonds (289.2 eV, 0.78%). Fig. 3b shows the O 1s XPS spectra of the GO film, which showed two components corresponding to the oxygen atom groups including O=C (531.8 eV, 85.6%) and O–C (533.4 eV, 14.4%). Fig. 3c–h show the ErGO films at different electrochemical reduction CV cycles, and the composition and ratio of the carbon and oxygen atoms are shown in Table 1. The ratio of the original GO film for carbon (C–C, sp^2^ and sp^3^) was 80.38%. The increase in the full width half maximum (FWHM) of the peaks in Fig. 3g show a clear trend. The main effect in XPS is a slight increase in the fraction of sp^2^ carbon atoms, however, the proportion of sp^3^ carbon atoms showed a dramatic increase.

The results showed that the ErGO film at CVs of 10, 50 and 100 cycles contained significant amounts of sp^2^ carbon atoms (78.59%, 79.31%, and 80.88%), signifying increases in most carbon atoms, but decreases in oxygen atoms on the GO due to the electrochemical reduction. This result showed that the C–C bonds were electrochemically reduced from GO sp^3^ to the structure of graphene sp^2^. In contrast, the C/O ratio of the GO film and that of the CV 100-cycle ErGO film were 4.1 and 31.57, respectively. Table 1 shows the C/O atomic ratios of GO before and after electrochemical reduction, which were obtained by analyzing the C 1s XPS spectral peaks. The C 1s XPS spectral peaks of the ErGO films yielded C/O ratios of 10.22, 16.92 and 31.57 after 10, 50 and 100 cycles, respectively.^1,7,10,37^

The relationship between the SPR angle and the XPS of the residual oxygen functionality of the ErGO film was further investigated. According to the XPS data, the C/O ratio of ErGO exceeded that of GO, confirming the effectiveness of electrochemical deoxygenation. This implied that the ErGO film from the electrochemical reduction process contained far less oxygen, thereby confirming the tunable band-gap and high quality of the ErGO.^37^

**Fig. 4:**
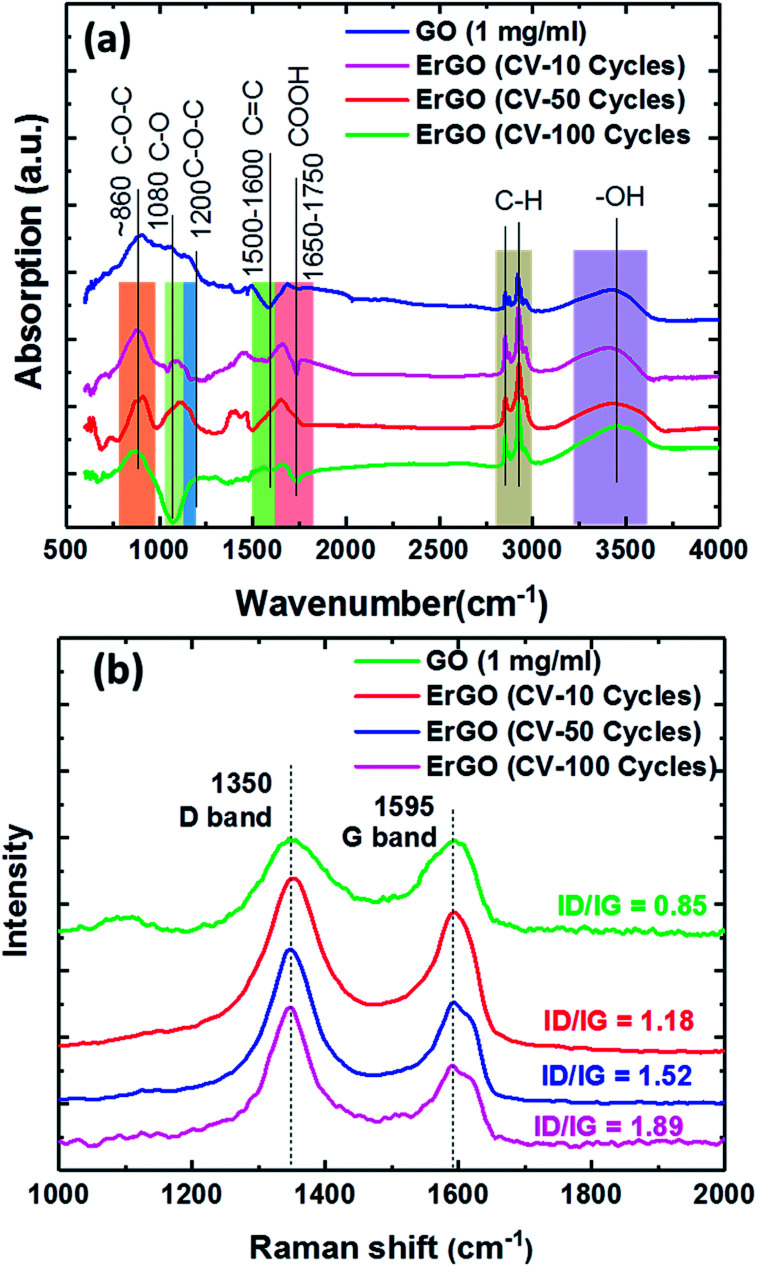
Analysis of 0.275 mg ml^−1^ GO films at different electrochemical reduction conditions for (a) FTIR spectra, and (b) Raman spectra of the GO films, showing D and G bands.

Fig. 4a shows the FTIR spectra of GO and ErGO films. The absorption peaks at approximately 860 and 1200 cm^−1^ were from the C–O–C of the epoxy stretching vibrations and the C–O of alkoxy stretching vibrations at approximately 1080 cm^−1^, respectively. The peak at around 1650–1750 cm^−1^ was caused by the carboxyl C=O stretching vibration of the COOH group. The peak O–H deformation vibrations in C–OH were seen at approximately 1305 cm^−1^, and the peak at around 1500–1600 cm^−1^ was attributed to the C=C skeletal vibration of the graphene sheets. The peak at around 2950–2850 cm^−1^ was attributed to C–H stretching vibrations due to pendant alkyl chains, and the peak at approximately 3410 cm^−1^ was due to –OH stretching vibrations. FTIR analysis showed an increase in C=C and decreases in C–O–C and O–H. Raman analysis of the same carbon lattice products revealed a G band, which represented the formation of the graphene sheet.

The sp^2^ carbon lattices were all common and produced by the stretching of C–C bonds. The GO peak near 1595 cm^−1^ was due to first order scattering of E2g phonons of the sp^2^ carbon atoms.^48^ However, whereas the D-band peak intensity revealed the plane vibrations attributed to the presence of the graphene structure defects,^49–51^ the G peak represented the ordered sp^2^ hybridization of the in-plane vibrations of the carbon–carbon bonds in graphene.^52^ The peak ratios of the intensity of the D and G peaks showed that rGO exhibited a significant increase compared to GO. The relative intensity ratio (*I*_D_/*I*_G_) is a measure of the defects present on a graphene structure. The results showed that the D-band was higher, meaning that sp^2^ bonds were broken, thereby resulting in more sp^3^ bonds. Therefore, the reduced GO had a higher *I*_D_/*I*_G_, meaning that there was a defect. The XPS showed a slight increase in the fraction of sp^2^ carbon atoms, however the proportion of sp^3^ carbon atoms increased dramatically. At the same time the D-band Raman intensity also increased, suggesting that the reduced GO had more defects than the original GO. This proved that the XPS and Raman were consistent with the sp^3^ carbon atoms being located at the defect sites, which is consistent with previous studies.^53–56^

Therefore, we analyzed the GO sheets under different electrochemically reduced conditions, which showed two characteristic Raman D and G bands at 1350 cm^−1^ and 1595 cm^−1^, respectively (Fig. 4b). The Raman spectra of the GO sheets showed the D/G intensity ratio (*I*_D_/*I*_G_ = 0.85). The ErGO sheets also contained both D and G bands in Raman spectra, with D/G intensity ratios of 1.18, 1.52, and 1.89 for 10, 50 and 100 CV cycles, respectively, which is larger than that of GO sheets (*I*_D_/*I*_G_ = 0.85). As a comparison, the electrochemically reduced ErGO sheets exhibited a much higher D/G intensity ratio of 1.89.

### 3.3 Analysis of PL emission spectra properties of the GO and ErGO solution

**Fig. 5:**
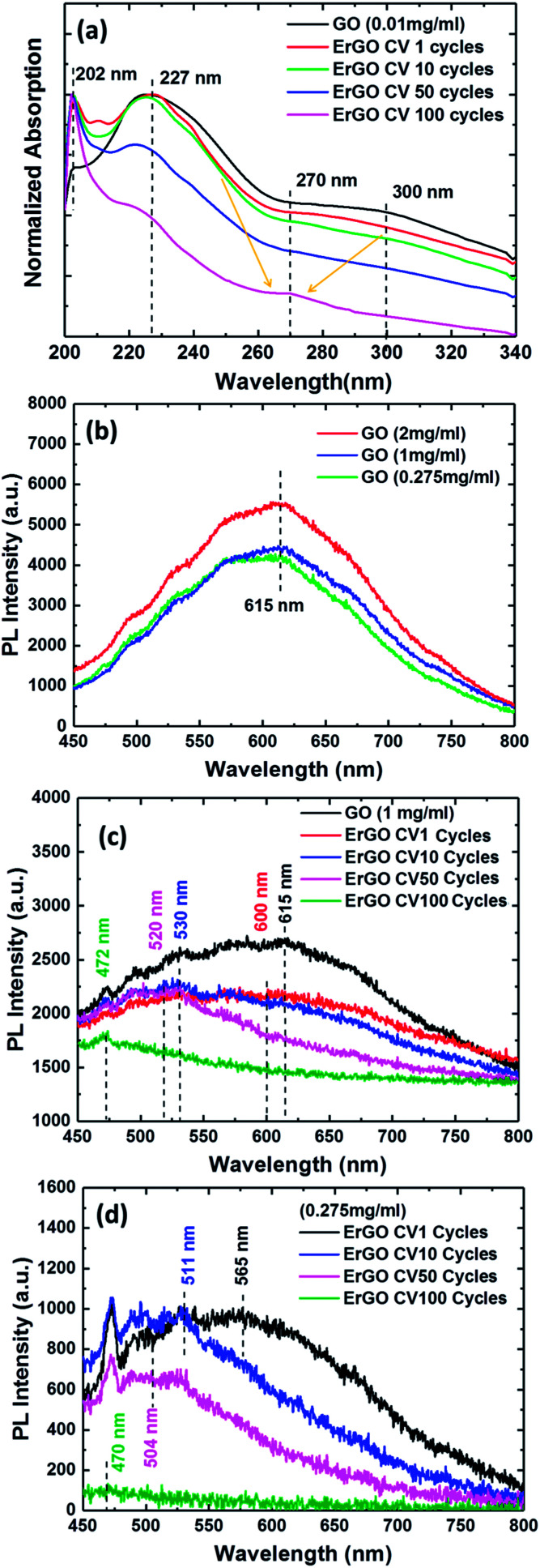
(a) UV-vis absorption spectra of the GO and ErGO sheets. PL spectra of the GO and ErGO sheets at different concentrations of aqueous solutions. (b) GO sheets, (c) 1 mg ml^−1^ ErGO sheets and (d) 0.275 mg ml^−1^ ErGO sheets at different electrochemical reduction conditions.

In order to investigate whether the formation of UV-vis absorption spectra of the GO and ErGO sheets could be stably dispersed in deionized water (DI water) solution, different CV cycle reactions were examined. Fig. 5a shows the UV-vis absorption spectra of the GO and ErGO sheets at a concentration of 0.01 mg ml^−1^ in suspension. We observed that the GO sheets typically had two absorption bands at approximately 227 nm attributable to the π–π* transition of the atomic C–C bonds, and 300 nm attributable to n–π* transitions of aromatic C–O and C=O bonds.^57^ In addition, with the increase in the number of reductions, the absorption peaks at 227 and 300 nm slowly disappeared, whereas ErGO from CV 1 to 100 cycles had a characteristic absorption band shift at approximately 270 nm, which corresponded to the π–π* transition of C–C bond shifts to 270 nm, indicating the reduction of GO and restoration of C=C bonds in the ErGO sheets.^58^

In this normalized PL measurement, the spectra showed GO and ErGO at different concentrations of aqueous solution and electrochemical reduction conditions (Fig. 5b–d). The PL optical behavior of the photo-excited electrons in GO and ErGO was due to non-radiative relaxation and radiative recombinations from discrete sp^2^-related states and continuous-defect states.^59,60^ GO consists of many disordered defect states within the π–π* gap and exhibits PL spectra with long wavelengths and broad optical frequencies. However, ErGO had a lower number of disordered inducible defect states in the π–π* gap and an increased number of clusters of newly formed small isolated sp^2^ domains.^59^ Therefore, the results showed that the PL spectra of GO and ErGO solutions at room temperature shown in Fig. 5b exhibited a broad PL response from 450 nm to 800 nm. Fig. 5b shows the PL spectra of GO solution at room temperature at concentrations of 0.275, 1, and 2 mg ml^−1^ with an excitation wavelength of 405 nm. It can be seen that the PL intensity tended to increase with increasing concentrations of the solution, which is similar to previous studies.^61–63^

Fig. 5c and d represent the PL spectra at *λ*_ex_ = 405 nm of rGO at four different reduction cycle conditions for solutions with concentrations of 0.275 and 1 mg ml^−1^, respectively. The rGO sheets exhibited quenching of PL emission spectra resulting in a blue-shift due to an increase in the number of sp^2^ clusters after reduction.^64^ Therefore, the tunable PL spectra during the reduction of GO could be attributed to changes in the relative intensities of PL emission of the two different types of electron excitation states. The PL emission spectra of ErGO due to the disappearance of functional oxygens atoms was due to restoration of more sp^2^ clusters, and the newly formed sp^2^ clusters in rGO could provide percolation pathways between the sp^2^ clusters already present.^64,65^ This result showed the reduction of the sp^2^ and sp^3^ hybridization of the GO and ErGO heterostructures.^59,60^

The pure GO sheets exhibited a PL band with the maxima at 615 nm, as shown in Fig. 5b. In contrast to the GO sheets, the ErGO sheets, with an increased number of electrochemical reduction cycles, showed a gradual shift in the spectrum to the blue band. Fig. 5c shows that the GO sheets at a high concentration of 1 mg ml^−1^ had electrochemical reduction condition indices of 1, 10, 50, and 100 CV cycles at center wavelengths of 600, 530, 520 and 472 nm, respectively, compared to 565, 511, 504, and 470 nm, respectively, for the 0.275 mg ml^−1^ GO sheets (Fig. 5d). The PL experiments showed the PL emission spectra for the direct transition types of GO and ErGO semiconductor materials,^14,18,59^ and confirmed that the real-time and stepwise deoxidization process of the electrochemical reduction of GO sheets could effectively tune the PL emission spectra.

## 4. Conclusions

We successfully demonstrated the absorbance and photoluminescence spectral features observed in different samples through real-time and stepwise processes in the reduction of GO. In contrast to the GO sheets, the ErGO sheets, with an increased number of electrochemical reduction cycles, showed that the spectrum gradually shifted to the blue band. The results of the photoluminescence emission measurements showed that the GO sheets had a peak wavelength at 615 nm. In addition, the electrochemical reduction condition indices of 1, 10, 50, and 100 CV cycles of GO sheets at 1 mg ml^−1^ showed peak wavelengths of 600, 530, 520 and 472 nm. The experimental results showed that the C 1s XPS spectral peaks from the ErGO films yielded C/O ratios of 10.22, 16.92 and 31.57 after 10, 50 and 100 cycles, respectively. The Raman spectra of the GO sheets showed an *I*_D_/*I*_G_ of 0.85, compared to 1.18, 1.52, and 1.89 for 10, 50 and 100 CV cycles, respectively, in the ErGO sheets. EC-SPR real-time monitoring of the band-gap and stepwise control of GO oxygen-containing functional groups are conducive to the future development of GO-based fluorescence materials and could increase their market potential. GO-based fluorescence biosensors have great potential due to their photophysical properties and sensing applications. GO-based fluorescence biosensors may therefore be beneficial for use in highly selective, rapid and low-cost assays, with promising economic output values.

## Conflicts of interest

The authors declare that they have no competing interests.

## Supplementary Material
